# Analysis of the Contribution of Wind Drift Factor to Oil Slick Movement under Strong Tidal Condition: *Hebei Spirit* Oil Spill Case

**DOI:** 10.1371/journal.pone.0087393

**Published:** 2014-01-30

**Authors:** Tae-Ho Kim, Chan-Su Yang, Jeong-Hwan Oh, Kazuo Ouchi

**Affiliations:** 1 Department of Ocean Environmental System Science, University of Science and Technology, Daejeon, Korea; 2 Korea Ocean Satellite Center, Korea Institute of Ocean Science and Technology, Ansan, Korea; 3 Department of Convergence Study on the Ocean Science and Technology, Ocean Science and Technology School, Korea Maritime and Ocean University, Busan, Korea; 4 Maritime Safety Research Division, Korea Research Institute of Ships & Ocean Engineering, Daejeon, Korea; University of Vigo, Spain

## Abstract

The purpose of this study is to investigate the effects of the wind drift factor under strong tidal conditions in the western coastal area of Korea on the movement of oil slicks caused by the *Hebei Spirit* oil spill accident in 2007. The movement of oil slicks was computed using a simple simulation model based on the empirical formula as a function of surface current, wind speed, and the wind drift factor. For the simulation, the Environmental Fluid Dynamics Code (EFDC) model and Automatic Weather System (AWS) were used to generate tidal and wind fields respectively. Simulation results were then compared with 5 sets of spaceborne optical and synthetic aperture radar (SAR) data. From the present study, it was found that highest matching rate between the simulation results and satellite imagery was obtained with different values of the wind drift factor, and to first order, this factor was linearly proportional to the wind speed. Based on the results, a new modified empirical formula was proposed for forecasting the movement of oil slicks on the coastal area.

## Introduction

Among maritime environmental disasters, oil spills in coastal waters seriously affect the ecological system, fisheries, and the economy. Although the number of large oil spills (>700 tons) has been decreasing, it is still a major problem for marine environments. Stranded oil tankers and collisions are the main causes of large oil spills, accounting for 64% of the total during the period of 1970–2010 [1]. Once oil is spilled from wrecked ships, it is essential to predict the movement of oil slicks as quickly as possible for appropriate actions to be taken to protect from and mitigate the damage. Since several early experiments using floating plastic cards and visual observation [2], [3], a substantial number of studies have been reported on predicting the movement of oil spills, including advection, spreading, dispersion and evaporation [4], [5], [6], [7], [8], [9], [10], [11], [12], [13]. These models are relatively complex, requiring exact information on the environmental conditions such as winds, currents, waves, turbulence, salinity, temperature, and solar insolation for the accurate simulation of movement of oil slicks. It appears, however, the model’s accuracy is still the subject of further research as pointed out by Mariano et al. [14]. Advection of oil slicks on the sea surface is generally defined, in its simplest form, by surface current associated with wave-induced current, tidal current and mean or tributary currents, and by wind-driven current. Note that the surface current here implies the integrated current at the surface layer, which carries oil slicks. From empirical data, the wind drift coefficient, i.e., the magnitude of wind-driven current induced by wind stress is approximately 0.03 of the wind speed at 10 m height and its direction can be assumed as nearly parallel to the wind direction [15], [16], [17]. Abascal et al. [18] used a set of drifting buoys during the 2002 *Prestige* oil spill in the Bay of Biscay, Spain to calibrate and validate the oil transport model together with meteorological data, indicating that the wind drag coefficient was linearly proportional to the wind speed under strong tidal currents, ranging from approximately 0.02 to 0.04 with the mean value of 0.027. Thus, the movement of oil slicks can be predicted, to first order, by this simple empirical formula if the current and wind fields are known.

Apart from forecasting the oil slick movement, remote sensing using airborne and spaceborne sensors is the most efficient technique for monitoring oil slicks on a regional, as well as global scale, and much effort has been made for accurate detection, identification and classification of oil-covered surfaces [19], [20], [21], [22], [23], [24]. In the recent cases, for example, a large number of airborne and spaceborne imagery have been acquired and analyzed for the 2007 *Hebei Spirit* oil spill in the Yellow Sea [25], [26], [27] and the aforementioned 2011 *Deepwater Horizon* oil spill [14], [28], [29], [30]. There are two types of remote sensing used for this purpose. One is optical remote sensing, utilizing the visible to infrared spectral band of the electromagnetic wave. The other is to use imaging radar. In the visible spectrum (approximately 400–700 nm) used by optical remote sensing, oil slick detection is based on its higher surface reflectance than seawater [19]. However, imagery is limited by cloud cover and only daytime observation can be made. SAR, on the other hand, has all-weather and day-and-night imaging capability [31]. The imaging mechanism of oil slicks by SAR is based on the difference in surface roughness. Oil slicks on the sea surface dampen short gravity-capillary surface waves, and thus, reduce the radar backscatter [21], [23], [27], [32], [33], [34], resulting in dark features in SAR images. Generally, the algorithm for detection of oil slicks in SAR images is based on the approach to distinguish the areas of small image intensity values by thresholding. The main problem is that it is difficult to identify the oil-covered surface from lookalike surfaces under low to no wind, rain cells, upwelling, and biogenic slicks, since the images of both surfaces appear dark with similar radar cross section. Several techniques have been proposed to identify oil slick features from lookalikes using image classification algorithms, such as those based on fuzzy logic, neural network, and polarimetric analyses [23], [24], [29], [31]. Each technique has pros and cons, but the movement of oil slicks cannot be predicted from SAR imagery or by optical data. Numerical oil transport models, on the other hand, are able to predict the oil slick movement accurately. Thus, it is expected that the combination of numerical simulation and remote sensing data should increase the accuracy of detecting, classifying, and forecasting oil slicks spilled on the sea surface.

In this paper, preliminary results are presented on the simulation of oil slick movement in the 2007 *Hebei Spirit* case. In the previous studies of this case, dispersion and advection of the oil spill were estimated using satellite data and ocean models [35], but a fixed wind drift factor was used in the simple empirical formula [36], resulting in limited performance in the initial transport of oil slicks. Furthermore, quantitative evaluation for the model accuracy was not made. This area in the west coast of Korea has characteristics of strong currents and seasonal variability of wind. Under these conditions, therefore, the fixed wind drift factor may cause some uncertainty in the resultant oil slick movement, and varying the wind drift factor may be able to better represent the distribution of surface oil slicks. The validation of the varying wind drift factor in the oil transport model using satellite imagery is indeed the main theme of this study. In the present study, the same simple empirical formula is used, requiring only the information on 2-dimensional current velocity and wind speed, and therefore, forecast of spatial and temporal distribution of oil slicks can be made with little information in real-time or near real-time. The results are then compared quantitatively with spaceborne optical and SAR data. Unlike the previous applications, the wind drift factor is varied almost linearly from 0.01 to 0.06 in order to find best fits to the satellite images.

The paper is organized as follows. In section 2, the *Hebei Spirit* oil tanker accident and satellite data are described, followed by the method of slick movement simulation in section 3, including the simulation model, current and tidal data and the results. In section 4, quantitative evaluation and discussions on the simulation accuracy are presented by comparing the oil slick distributions simulated by both the models with a fixed and varying wind drift factors with the satellite data, and conclusions in section 5.

## *Hebei Spirit* Oil Tanker Accident and Satellite Data

### *Hebei Spirit* Accident

**Figure 1:**
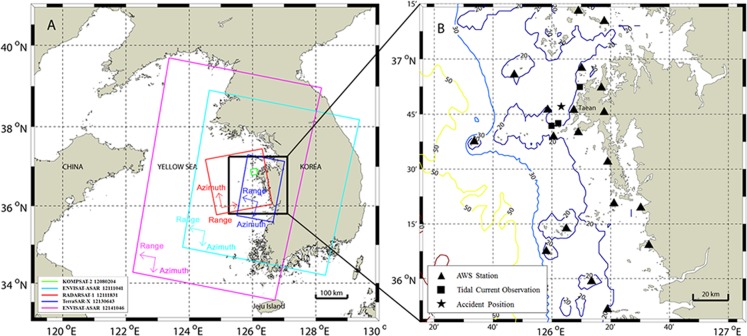
Coverage map of satellite data and information on the study area. Coverage of satellite map and study area around the 2007 *Hebei Spirit* oil tanker disaster. (A) Coverage map of acquired satellite data from 8^th^ to 25^th^ December, 2007. The smallest green box is the coverage area of KOMPSAT-2 optical sensor, and other boxes are those by SARs as indicated in the legend. ENVISAT ASAR and TerraSAR-X were in descending orbit and RADARSAT-1 in ascending orbit. (B) Bathymetry and positions of Automatic Weather System (AWS) stations and tidal current observation.

On December 7, 2007 at local time (LT) 07∶15 (UTC 22∶15, December 6), the oil tanker *Hebei Spirit* collided with a crane vessel about 8 km off the coast of Taean, on the west coast of Korea (approximate location: 36° 49.93′N, 126° 2.46′), spilling approximately 10,900 tons of crude oil [26]. The oil spill was stopped at about 07∶30, December 9 (LT). The location of the affected area is shown in Figure 1b. At the time of collision, the sea state was high with wind speed and significant wave height of approximately 14 m/s and 3–5 m, respectively (high sea state was one of the reasons for the collision). Due to strong wind and currents at the incident site, some of the spilled oil reached the nearby shoreline 17 hours after the accident, and some spread over the open sea. 9 days after the accident, 167 km along the south coast in Taean was contaminated by crude oil, and oil slicks also polluted a huge area in the open sea, including near Jeju Island, located at the southern end of the Korean Peninsula.

### Study Area

In the eastern Yellow Sea, surface current is dominated by tidal current strongly formed by high tidal range [37]. The tidal current is one of the most energetic oceanic components in the Yellow Sea, and suspended sediment transport processes are strongly affected by strong tides [38], [39]. The sea around the Taean Peninsula on the west coast of Korea is shallow (<40 m) as shown Figure 1b. In the western area of Taean, the relatively flat bottom topography is formed at the water depth of approximately 25 m. The Korea Hydrographic and Oceanographic Administration (KHOA) provide tidal range and currents in the coastal areas of Korea (http://www.khoa.go.kr/). In the waters of the polluted area, the tides are semi-diurnal (M2, S2 are larger than other components), with a tidal range (spring tide: 5.73 m, neap tide: 2.86 m) and move in a southwest direction. Tidal currents are approximately 0.1–1.6 m/s and the directions are northeast and southwest when flood and ebb current, respectively. Maximum flood is 1.52 m/s and maximum ebb is 1.65 m/s. Wind has a clear seasonal variability, because Korea is located in the seasonal wind area of northeast Asia. In spring and summer seasons, south/southwestern winds blow, and north/northwestern winds in autumn and winter season are typical seasonal winds. In particular, at the time of the accident in December, there was strong persisting north/northwestern wind.

### Satellite Data

**Figure 2:**
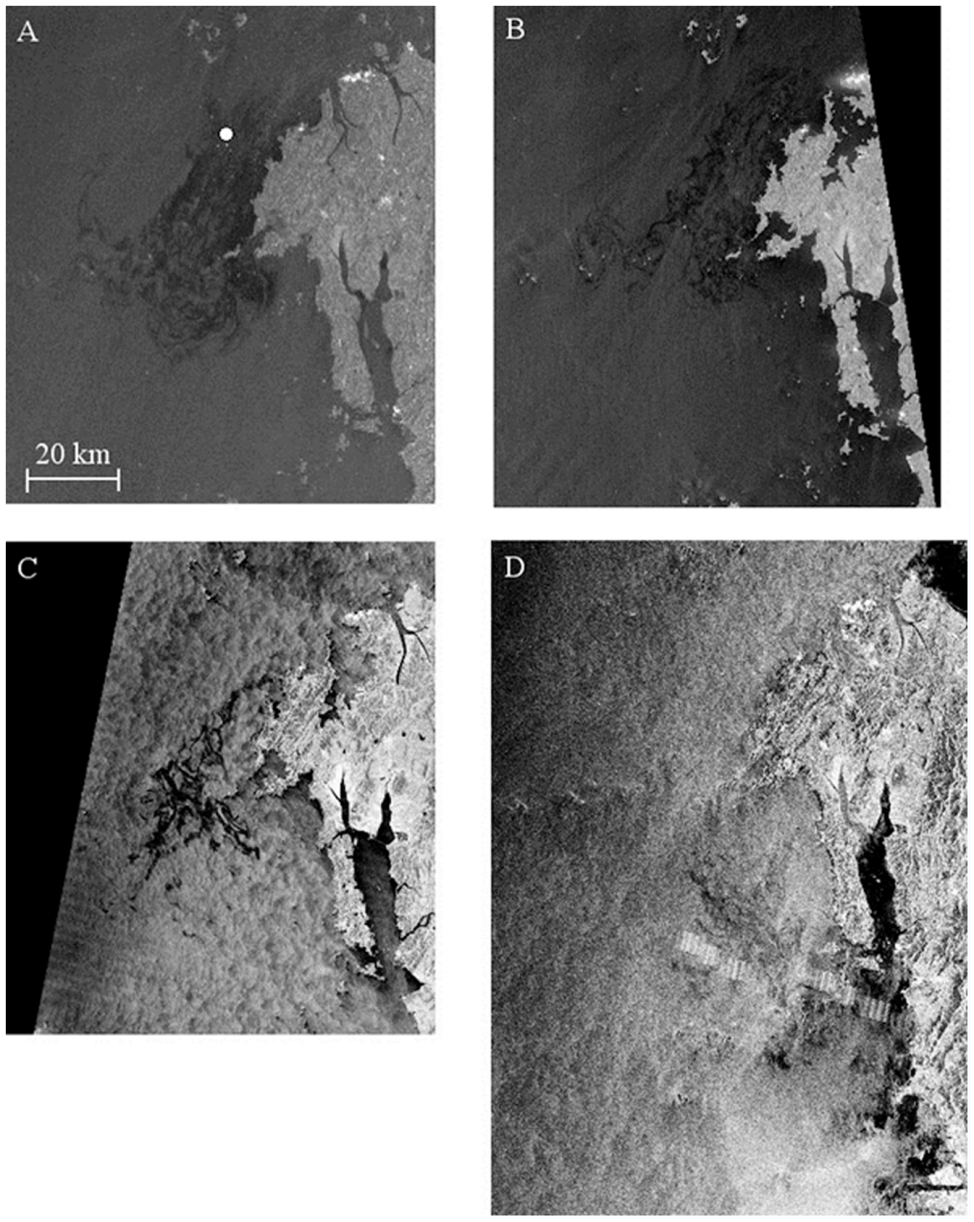
SAR images of oil slicks. SAR images of oil slicks acquired by different times as listed in Table 1, where (A) ENVISAT-ASAR, (B) RADARSAT-1, (C) TerraSAR-X, and (D) ENVISAT-ASAR. The position of the *Hebei Spirit* was marked by the white circle in (A).

After the incident, several images were acquired using spaceborne platforms from December 8^th^ to 25^th^, 2007. In this study, an optical image acquired by KOMPSAT-2 MSC (multi-spectral camera) and 4 SAR images acquired by ENVISAT-ASAR, RADARSAT-1, and TerraSAR-X were used for comparison with the simulation results. Figure 1a illustrates the coverage areas, and Table 1 shows the data acquisition time, sensor mode, polarization, swath width, spatial resolution, wind direction, and wind data at the Automatic Weather System (AWS) stations closest to the centroid of the slick areas at each satellite data acquisition time. Among 5 sets of satellite data, the KOMPSAT-2 image is not shown here as it was already given in the previous paper [25] with detailed analysis. Briefly, the KOMPSAT-2 MSC has high spatial resolution of 1 m for panchromatic data and 4 m for multispectral (4 bands) data between 450–900 nm with the swath width of 15 km. In the study, multispectral images were used by applying geometrical correction with reference to aerial optical photographs. The oil-covered areas were then extracted by thresholding the image brightness. For SAR data, the radiometric calibration was first made to calculate normalized radar cross section (NRCS), and geometric correlation was also applied to match the KOMPSAT-2 image and aerial photographs. Figure 2 illustrates the geocoded SAR images acquired at 4 different times.

**Table 1:** Parameters of satellite sensors and wind data at the AWS stations closest to the centroid of the slick areas at each satellite data acquisition time.

Satellite	KOMPSAT-2	ENVISAT ASAR	RADARSAT-1	TerraSAR-X	ENVISAT ASAR
**Acquisition time (LT)**	2007.12.08 11:04	2007.12.11 10:40	2007.12.11 18:31	2007.12.13 06:44	2007.12.14 10:45
Mode (*Sensor)/Polarization	*Multi-SpectralCamera (MSC)	Wide Swath/VV	Wide/HH	ScanSAR/VV	Wide Swath/VV
**Incidence angle [deg]**	-19.92(roll tilt)	31.0–36.3	31–39	31.8–40.5	19.2–26.7
**Swath width [km]**	15	405	150	100	405
**Nominal resolution [m] (range/azimuth)**	4/4	150/150	25/27	18.5/18.5	150/150
**Wind speed [m/s]**	4.8	5.6	6.3	4.3	6.9
**Wind direction [degrees]**	263.1	338.0	347.0	323.1	339.0

**Figure 3:**
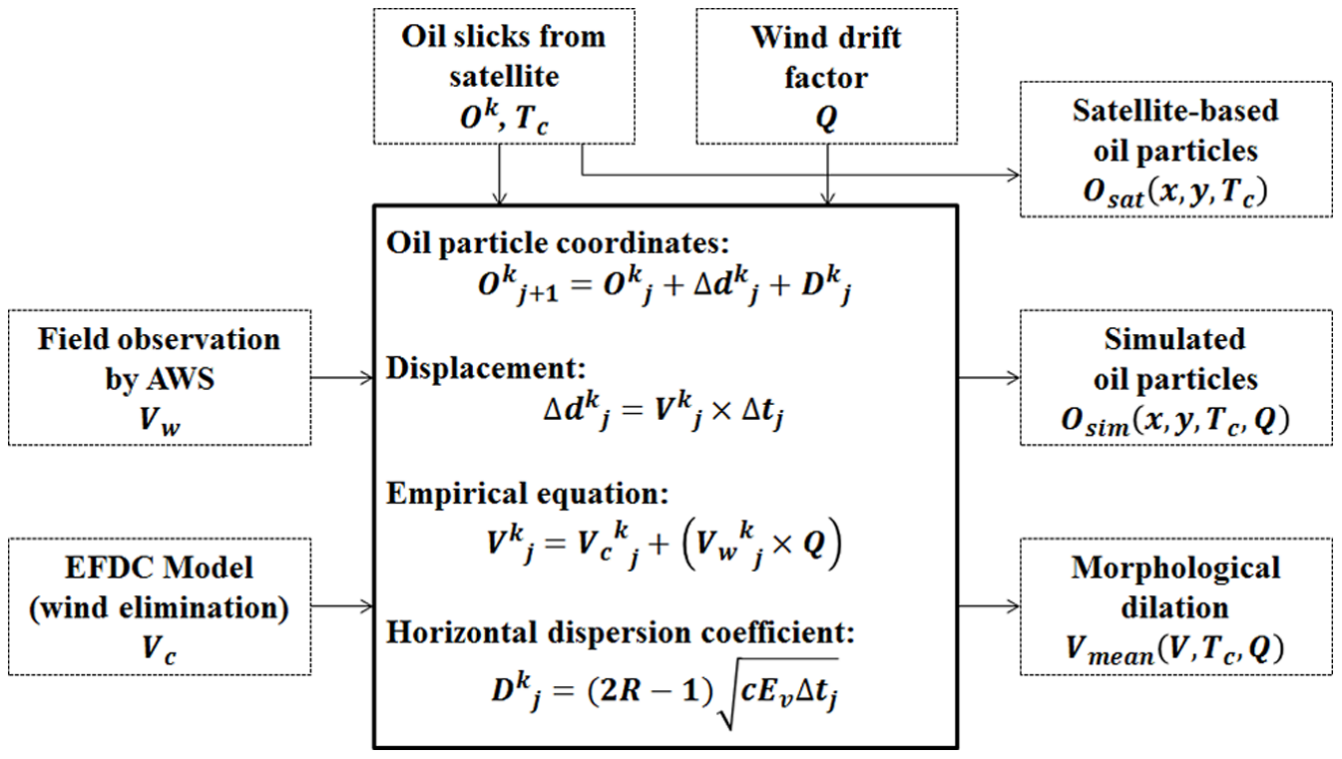
Flowchart for the numerical simulation of oil slick movement. Each parameter represents vector or scalar and explained in detail in the text. The central box represents equations for each step in the simulation.

As in the figure, the oil slick spread during 4 days after the collision in a southward direction from the source of the oil spill, which is indicated in a white circle in Figure 2a. Substantial changes in the slick pattern can be observed at only 8 and 36 hours of time difference between Figures 2a and 2b, and between Figures 2b and 3c respectively. 7 days after the accident on the December 14^th^, a certain amount of spilled oil appears to have dissipated, and some reached further south as can be seen in Figure 2d. It should be noted that the dark areas in the inland sea and south coast of the Taean Peninsula seen in Figures 2b, 2c and 2d were wind-sheltered areas, but not covered by oil slicks. As mentioned previously, it is difficult to distinguish wind-sheltered lookalikes and oil-covered surfaces by SAR without *in-situ* data or appropriate processing methods of image classification.

The threshold values were computed using a numerical scattering model depending on the radar parameters, oil properties, and wind related to each SAR image [27]. They were used to distinguish region of interest (ROI), which includes oil slicks and lookalikes. Oil slick areas were determined by the histograms of NRCS in ROI and visual inspection on the basis of reports from Central Disaster and Safety Countermeasures Headquarters. The positions of extracted slick areas were then coordinated at grids of a regular interval, and used as initial conditions in the particle tracking simulation. That is, the slick images extracted from the KOMPSAT-2 image were used to simulate the slick areas in the first ENVISAT-ASAR image, which in turn was used to simulate the slick areas in RADARSAT-1 image, and so on. To simulate the slick areas observed in the KOMPSAT-2 image, oil discharge from *Hebei Spirit* was assumed as described further in the following section.

## Simulation of Oil Slick Movement

### Oil Slick Transport Models

The pioneering works on modeling the movement of oil slicks were made by Fay [4], [5] and Hoult [6]. The model of Fay considers an oil slick in calm waters spreads circularly in three phases. In the first phase called the gravity-inertial spread, in which the radius of slick is determined by the inertial force of initially spilled thick oil, which is balanced by gravity, and in the second stage of the gravity-viscous spread, the thickness of oil starts to decrease and the time dependence is determined by the balance of the forces due to viscosity and horizontal gravitational pressure. In the final stage of surface tension, the oil slick becomes very thin, and the time dependence of the radius is determined by the equal forces due to viscosity and surface tension. Although the equations developed by Fay [4], [5] and Hoult [6] cannot fully explain oil spreading, they are the basis of spreading algorithms used today. Since these early studies, more realistic models have been developed by including the oil movements caused by currents and winds, and the fate processes such as evaporation, emulsification, and dissolution [4], [5], [6], [7], [8], [9], [10], [11], [12], [13] [14]. Currently, there exist several operational models, including General NOAA Operational Modeling Environment (GNOME) [40], the ensemble forecast system of university of South Florida [41], IMO model [36], and those by Castanedo et al. [41] and Brostrom et al. [42].

In general, oil tracking by numerical models uses virtual particles [10]. These particles seeded in the models are advected with surface velocity field, dissipated by the fate processes. Among many simulation models, Korotenko et al. [12] developed a hybrid algorithm consisting of a transport and hydrodynamic models. The model is based on the Lagrangian particle tracking algorithm [43] which includes the effects of surface evaporation and decay [44]. The algorithm in its principal form is similar to that used in this study, i.e., oil particles are transported by the mean and wind-driven current (with a constant drag coefficient) and random diffusion, but in 3-dimensions and includes the effects of evaporation, emulsification, and decomposition by applying the lifetime on oil particles, which was empirically derived and dependent on several factors such as types of oil and thickness. The model is, therefore, rather complex requiring much information on spilled oil and environment. The transport model was implemented to predict the oil slick movement in the Caspian Sea, but comparison with real data was not made and its accuracy was not validated. Dietrich et al. [28] used, without the fate processes of Korotenko et al. [12], a simple numerical model based on the mean current, wind-driven current, and stochastic dispersion to simulate the 2011 *Deepwater Horizon* oil spill in the Gulf of Mexico where the tidal variation is very small. The current velocity field was computed using SWAN (Simulating Waves Nearshore) +ADCIR (Advanced CIRCular) models [45]. They used three different wind drift factors (0.00, 0.01, and 0.03), and the comparison with satellite data showed that the best agreement was obtained with wind drift factor of 0.00. Our simulation model described in the following subsections is similar to that of Dietrich et al. [28], but with varying wind drift factors and in the Yellow Sea where the tidal variation is very strong.

### Simulation Model

Numerical simulation for the prediction of oil slick movement uses virtual particles which represent the locations of oil slicks extracted from satellite images [10], [14], [25], [28]. To determine the coordinates of virtual particles, a lattice cell search approach was used. In this search, each image was overlaid with lattice cells whose size ranged from 10 to 850 m depending on the spatial resolution of the images. The cells containing oil slick are extracted, and defined as virtual particles. These particles are then advected by the surface current of which the velocity can be expressed by the following simple equation as a sum of tidal current and wind-induced current velocities [36].
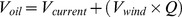
(1)where *V_oil_* and *V_current_* are the velocities of the oil slick and tidal current respectively, *V_wind_* is the wind speed at a height of 10 m, and *Q* is the wind drift factor. As in Equation (1), the wind-induced current velocity is expressed in terms of the fraction *Q* of wind speed along the current direction [45], [46], [47]. The wind drift factor can range from 0.01 to 0.06; [11], [18], [48], but in many applications of oil movement forecast, *Q* is fixed as 0.03 [8], [9], [36]. In the present study, Equation (1) in the 2-dimensional vector form is used to compute the spatial and temporal changes of oil slicks with *Q* as a variable. Note that the model does not take account of the effects of oil thickness, dispersion, spreading, evaporation and weathering, but it takes account the breaching of oil spills, i.e., if an oil particle reaches the coastline, it will be fixed at that position.

Figure 3 shows the flowchart for the simulation of oil slick movement, where *O^k^_j_* and *Δd^k^_j_* represent the coordinate and displacement of a virtual oil particle, respectively. *T_c_* and *Δt_j_* are the acquisition time of the satellite image and time step for particle displacement respectively. *V^k^_j_* is the surface current velocity computed from the empirical formula with wind and tidal current inputs. *k* ( = 1,2,…, *N_p_*) is the number of oil particles and *j* ( = 1,2,…, *N_t_*) is the number of time steps. The time step of simulation is 30 minutes. *V_w_* and *V_c_* represent the wind and tidal current velocities respectively, and *Q* is the wind drift factor. *D^k^_j_* is the horizontal dispersion coefficient having the unit of distance, *R* is a random number in the interval between 0 and 1, *E_v_* ( = 10 m^2^/s) is the turbulent coefficient, and c ( = 12) is a scaling coefficient [28]. Oil movements on the sea surface are affected by a random walk procedure which defined the horizontal turbulent diffusion. This stochastic velocity can be described by a two-dimensional diffusion coefficient. Consequently, the particle movement is defined in terms of the particle velocity and the horizontal dispersion coefficient. *O_sat_* and *O_sim_* in Figure 3 describe the extracted oil particles from satellite imagery and simulation results respectively at the end time *T_c_* and position (*x*, *y*). *V_mean_* is the mean surface current velocity during the start to end times of each simulation case (see section 3.5 for further detail) in each grid, which will be used to dilate the simulated slick images by morphological image processing for quantitative evaluation in section 4. As in Figure 3, the required parameters in this simulation are only the current and wind fields.

### Tidal Current Data

**Figure 4:**
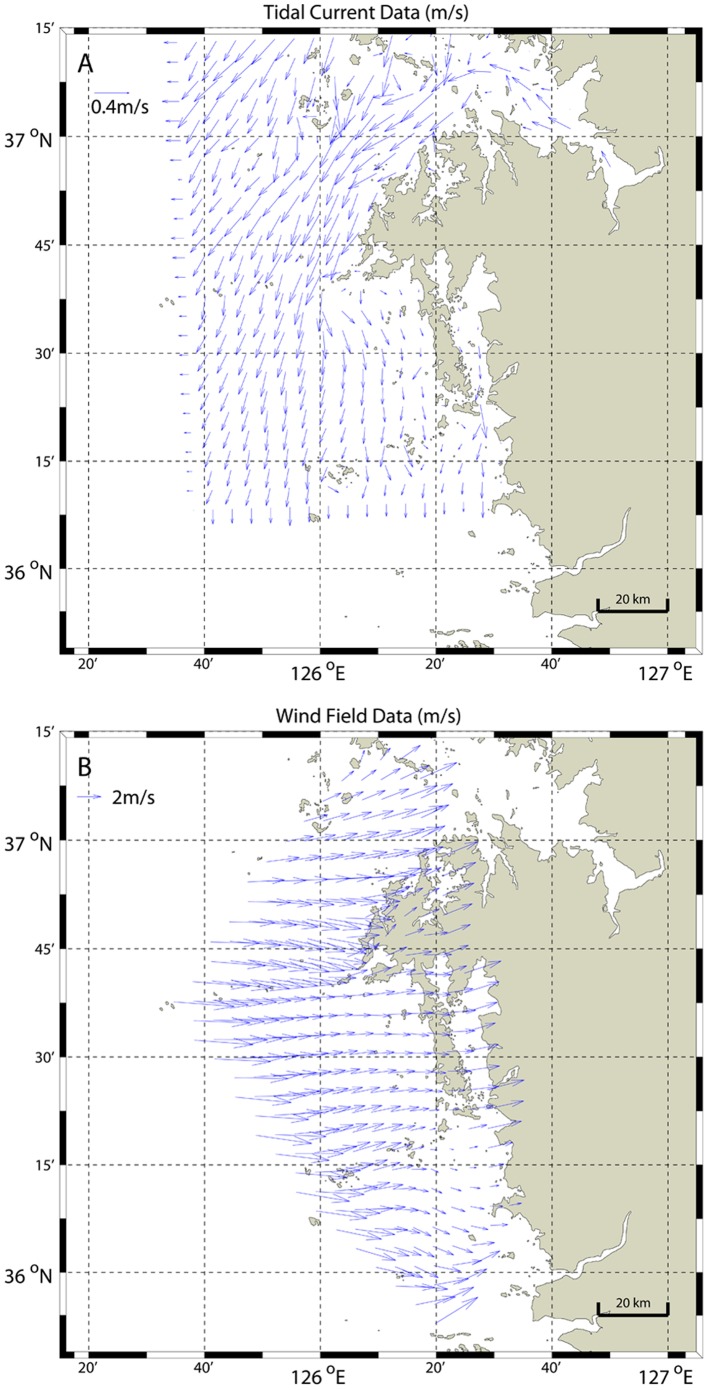
Tidal current and wind field around the oil slick area. Examples of tidal current and wind fields around oil slick area at 10∶40 on December 11, 2007. (A) tidal current data from EFDC without wind-induced current during spring/low tide and (B) wind field data from AWS.

**Figure 5:**
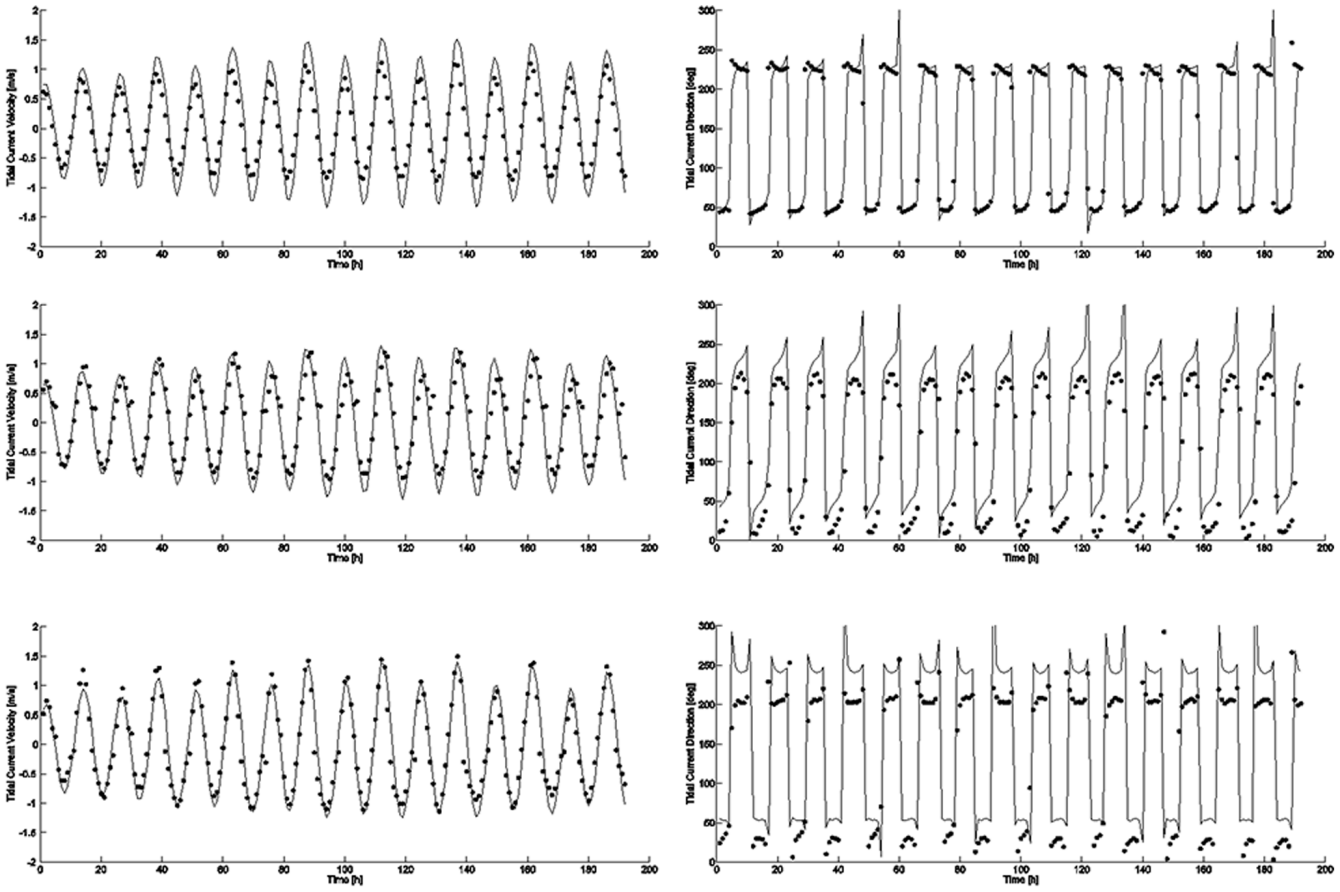
Comparison of EFDC and KHOA current data. Left and right columns show the comparisons of current velocity and direction respectively. The solid lines and dots correspond respectively to Environmental Fluid Dynamics Code (EFDC) and Korea Hydrographic and Oceanographic Administration (KHOA) data.

The coastal areas of the Taean Peninsula are vast tidal flats having high tidal range. The Environmental Fluid Dynamics Code (EFDC) [49] is used to compute the tidal currents. This model is a 3-dimensional hydrodynamic model based on continuity, momentum, salt balance, and heat balance with hydrostatic and Boussinesq approximations and used orthogonal curvilinear horizontal and vertical sigma coordinates. The model developed at the Virginia Institute of Marine Science (VIMS) and recommended by U. S. Environmental Protection Agency (EPA) for environmental applications. The EFDC model has been widely used in studying coastal and estuarine hydrodynamics and tidal flow. Yang et al. [25] estimated the tidal current fields in this area using the EFDC model with grid spacing of 500 m, as shown in Figure 4a, where the current velocity and direction at each grid were computed by interpolation and resampling the original data with the method of nearest neighbor. These grid data were used together with tidal data estimated at appropriate times required for simulation corresponding to each satellite image. The tidal current velocity and direction estimated by using the EFDC model were compared with those provided by KHOA. KHOA generates time series current data based on the *in-situ* measurements made at the three KHOA stations indicated by the black squares in Figure 1b. The results from 7^th^ to 14^th^ of December, 2007 are shown in Figure 5. Although there is small difference, fairly good agreements in both the current velocity and direction can be seen between the EFDC model and KOHA data. Further, the Nyquist period is the half the semi-diurnal period, which is approximately 6 hours 13 minutes. The time step of 30 minutes, therefore, well satisfies the sampling theorem, and represents the current variation used in the simulation. Note that Liu et al. [30] used a much longer time step of 3 hours for their simulation of the *Deepwater Horizon* oil spill due mainly to small tidal variation in the Gulf of Mexico.

### Wind Data

The Korea Meteorological Administration (KMA) deployed a high-density network of AWS over South Korea to collect real-time observations of surface meteorological parameters including temperature, wind speed and direction, pressure and rainfall (http://203.247.66.10/weather/observation/aws_table_popup.jsp). Wind data were obtained from 19 AWS stations at different heights around the position of collision. The data contain the wind speed and direction averaged over 1 minute at 1 minute intervals. Hellman exponential law was used to convert the measured wind speed to the wind speed at 10 m height, and it is defined as follows [50]:
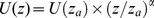
(2)where *U*(*z*) is the wind speed at height *z*, and *U*(*z_a_*) is the wind speed at height *z_a_*_,_ which is referred to as the 10 meter height in this study. *α* is the friction coefficient or Hellman exponent which is a function of the topography and roughness at a measurement site. If the site characteristics are yet to be determined, a value of 0.14 is a good first approximation [50], [51]. This value was used to convert the wind speed at 10 meter height, and the wind speed and direction were averaged during 30 minutes at each AWS station. Wind field at grid spacing of 500 m was generated by the same as for the current data using linear interpolation and the method of nearest neighbor. Figure 4b shows the wind velocities computed using this approach.

### Tidal Current and Wind Data during Hebei Spirit Oil Spill

**Figure 6:**
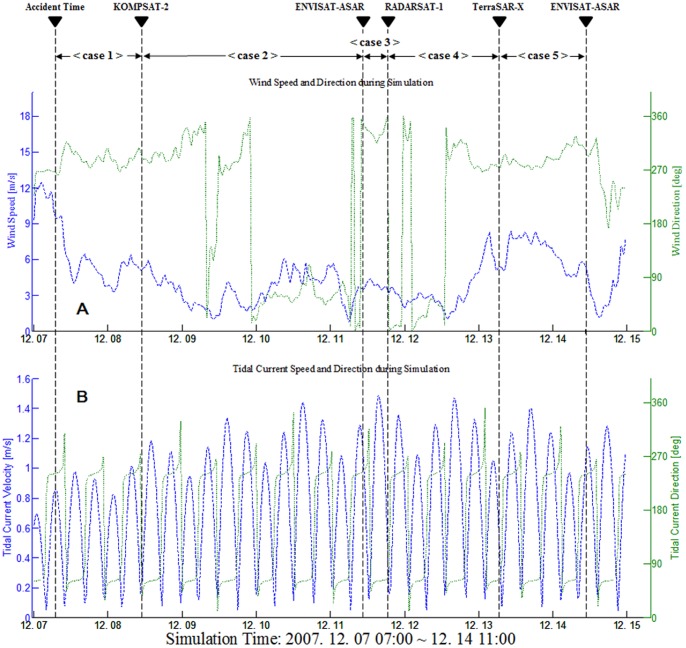
Average wind and tidal current during simulation period. Mean wind and tidal current averaged over 30 minutes at the position of *Hebei Spirit*. (A) represents wind speed (blue line) and direction (green line), and (B) represents tidal current velocity (blue line) and direction (green line). Vertical dashed lines represent the accident time and satellite data acquisition times.

The tidal current and wind fields at the *Hebei Spirit* collision position during the entire simulation period are shown in Figure 6. As can be seen from the figure, at the time of the accident the tidal current velocity was small but the wind speed was high. The tidal current velocity oscillated 4.5 cycles at the time of the accident and the KOMPSAT-2 data acquisition time with varying wind speed was approximately 3–9 m/s in the directions between 268 and 317 degrees. The current direction varied substantially, ranging from 30 to 270 degrees. Between the times of data acquisition by KOMPSAT-2 and ENVISAT-ASAR, tidal currents varied around 12 cycles with periodic directional changes of 6 cycles, while there was only a single cycle of current velocity and direction during the ENVISAT-ASAR and RADARSAT-1 data acquisition times. During the next period between the RADARSAT-1 and TerraSAR-X data acquisition times, the tidal cycles were similar to those of the first period, but the wind variation was large. In the last period between the TerraSAR-X and ENVISAT-ASAR data acquisition times, the tidal cycle was 3.5 times but the wind was strong, ranging from 5 m/s to over 8 m/s in the same approximate direction. These changes of tidal and wind fields resulted in the oil slick patterns varying in complex forms as observed in Figure 2.

### Simulation and Results

Simulation of particle movement was carried out for the 5 cases. In case 1, oil particles from the grid corresponding to the position of *Hebei Spirit* were generated at 30 minute intervals, starting from the time of the accident (at local time 07∶15, December 7^th^) until the time of the KOMPSAT-2 data acquisition. Using Equation (1) with the tidal and wind-induced current data, the particles were tracked until the time of the KOMPSAT-2 data acquisition as listed in Table 2, i.e., at local time 11∶04, December 8^th^. The locations of virtual particles were simulated for comparison with the satellite image.

**Table 2:** Start and end times of simulation for the cases 1–5.

Simulation case	Start time (at local time: UTC +9)	End time (at local time: UTC +9)	Time difference
Case 1	2007. 12. 07 07:15	2007. 12. 08 11:04	27 hrs 49 min
Case 2	2007. 12. 08 11:04	2007. 12. 11 10:40	71 hrs 24 min
Case 3	2007. 12. 11 10:40	2007. 12. 11 18:31	7 hrs 51 min
Case 4	2007. 12. 11 18:31	2007. 12. 13 06:44	36 hrs 13 min
Case 5	2007. 12. 13 06:44	2007. 12. 14 10:45	28 hrs 01 min

Start and end times correspond to the data acquisition times (local time) of each satellite, except the start time of the case 1 being the time of *Hebei Spirit* accident.

In the second case, the oil particles extracted from the KOMPSAT-2 image were used as the initial data, and tracked to simulate the slick areas in the ENVSAT-ASAR image acquired at local time 10∶40, December 11^th^. Since the oil discharge continued until 07∶30, December 9^th^ (LT), virtual particles from the wrecked tanker were also generated, tracked until the time when the discharge was ceased, and added to the simulated data. In the same way, the ENVISAT-ASAR image was used as the initial data to simulate the RADARSAT-1 image. This process was repeated for the simulation of particle movement for the rest of cases. The start and end time of all cases are listed in Table 2.

**Figure 7:**
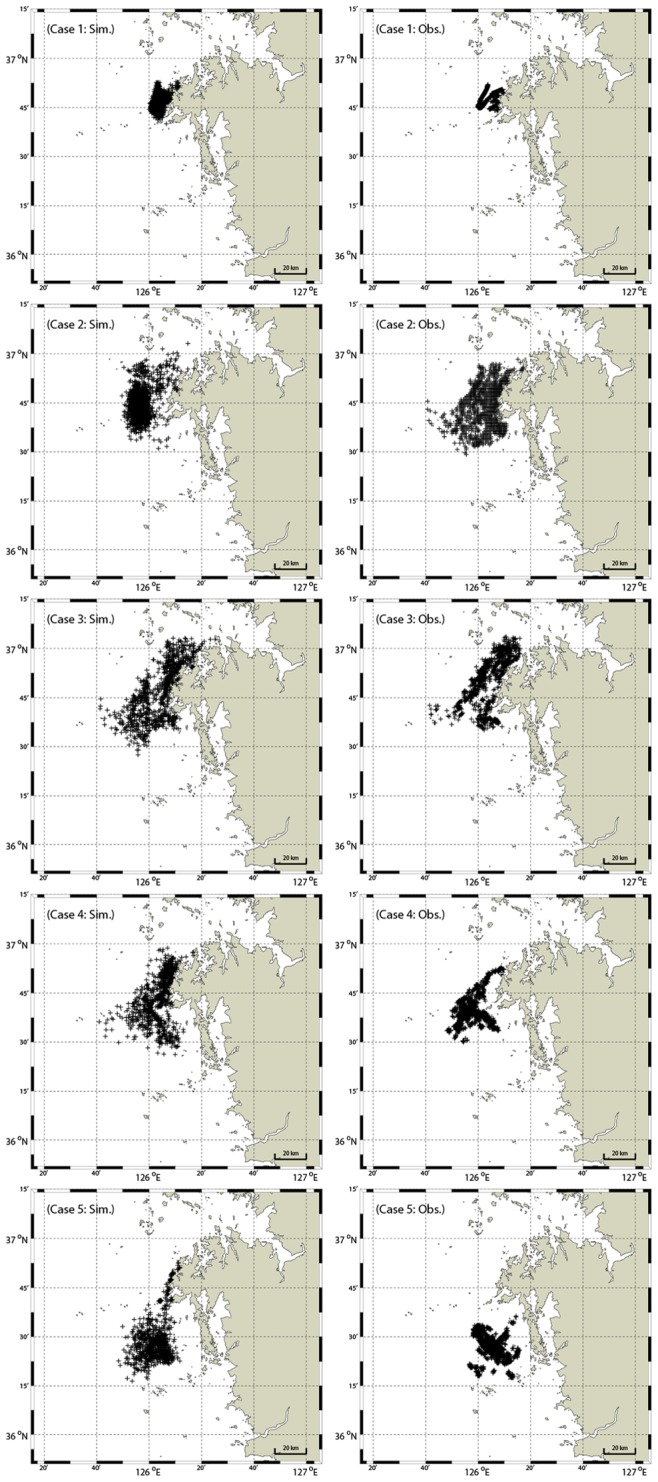
Comparison of oil slick areas by satellite images and simulation. Comparison of oil slick areas extracted from satellite images on the right column and those by simulation on the right column. From top to bottom rows: cases 1–5. See the text for further details.

For each cases of simulation, slick areas were computed using oil particles for different wind drift factors, ranging from 0.01 to 0.06 at intervals of 0.001, i.e., 51 sets of simulation data for each case, to be compared with the satellite data. The simulated oil particles are shown on the left column of Figure 7. The images are those which fit best, among 51 sets of each simulation case, to the observed satellite oil particles shown on the right column.

It can be seen from the figure that fairly accurate correlation was obtained between the simulation and KOMPSAT-2 data in case 1; while correlation was not as accurate in case 2, underestimating the slick areas by simulation in comparison with the ENVISAT-ASAR (on Dec. 11^th^) data. Correlation is fairly accurate for the rest of the cases, but the simulation results tend to overestimate the satellite data. In order to assess the accuracy of the simulation results shown in Figure 7, further quantitative analyses are carried out in the following section.

### Quantitative Evaluation and Discussion

**Figure 8:**
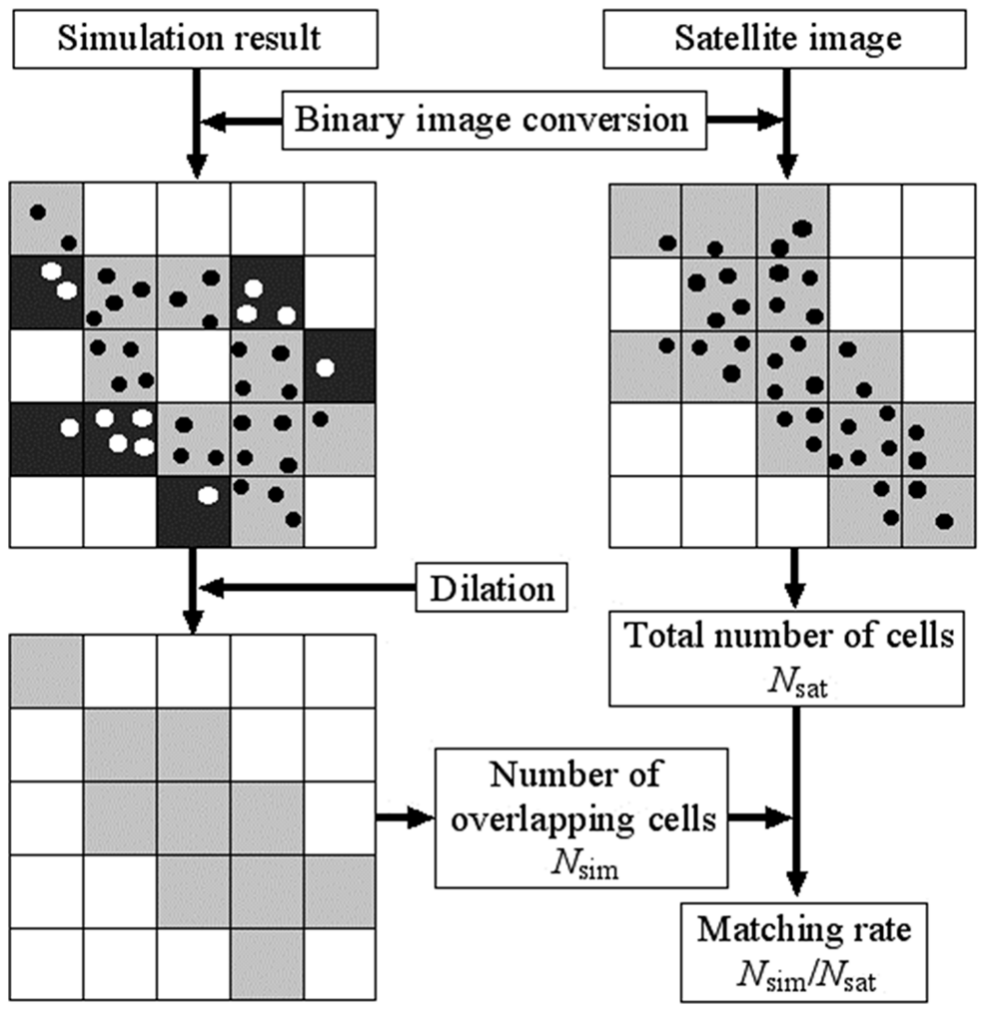
Diagram for quantitative evaluation of simulation results. Cells with light gray color in the simulated binary image indicate those occupied by oil particles that overlap with the cells containing oil particles in the satellite image, and cells with dark color are those occupied by oil particles but do not overlap with the oil cells in the satellite image.

In practical applications, oil slick movement is forecasted in a coordinate system composed of lattice cells or mesh [28]. In the present study, the same cell size as the current and wind fields was used, i.e., 500×500 m, to convert to binary image. In the evaluation procedure shown in Figure 8, cells containing virtual particles in the simulated binary image are first sought. These cells are indicated by dark and light gray colors in Figure 8. Then, the cells are selected, which overlap those of the satellite binary image containing oil particles. The overlapped cells in the simulated image are indicated by light gray color; while the non-overlapping cells are shown in dark gray color. In the next step, simulated virtual particles are dilated using structural elements in morphological image processing [52] as follows. During simulation, a particle moves along paths with velocity defined by Equation (1). The mean velocity vector (mean velocity and direction) *V_mean_* of this particle is computed and its initial point is assigned to the position of the particle at the end of simulation. If a cell at the terminal point of the vector *–V_mean_* is a non-overlapping cell, then this cell is regarded as an overlapping cell, which is illustrated in Figure 8 (the cell at the center). If the vector *–V_mean_* passes through multiple cells, these cells are also considered as overlapping cells. Physically, this process means that the cells, through which a virtual oil particle has passed with a mean velocity at a mean direction during the simulation period, are covered by oil slick. The matching rate is defined as the number of overlapping cells in the simulated image *N_sim_* divided by the number of cells in the satellite image *N_sat_*.

**Figure 9:**
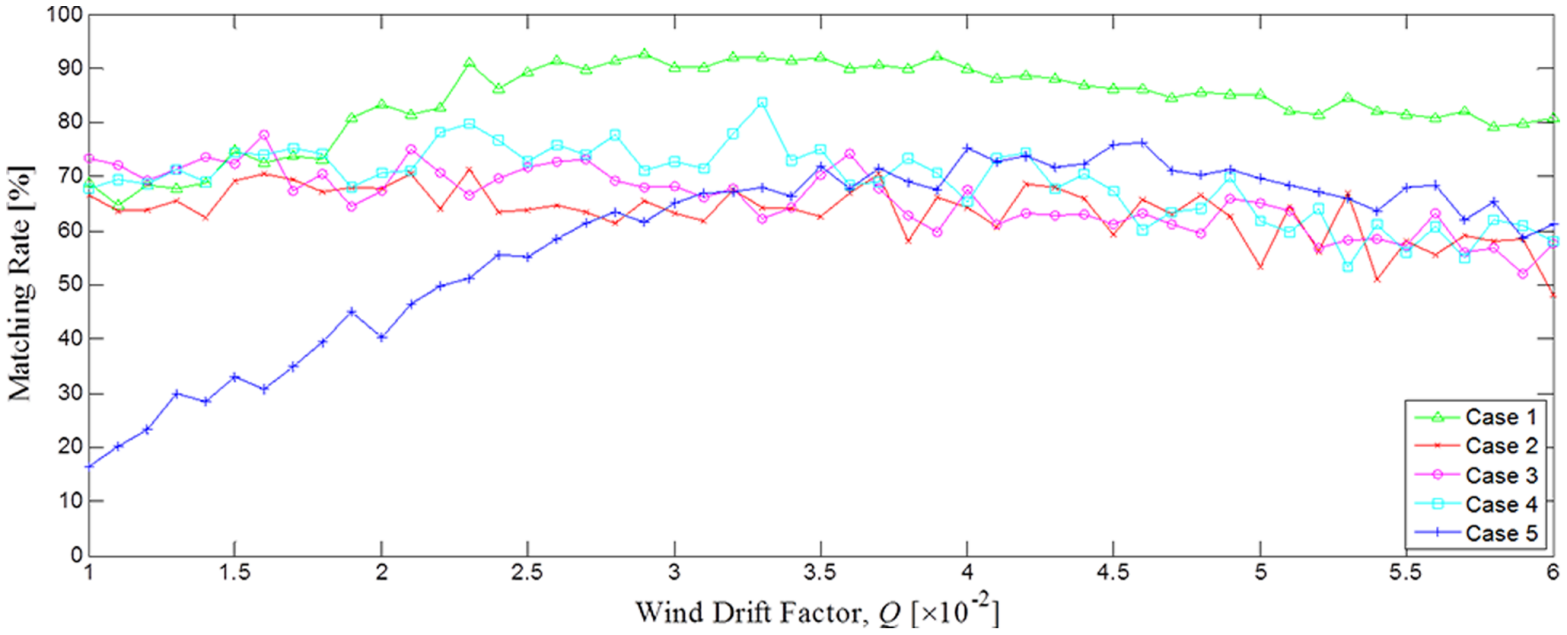
Matching rates as a function of wind drift factor. Matching rates of the simulated oil slick areas compared with those of satellite images as a function of different wind drift factor for cases 1–5.

**Figure 10:**
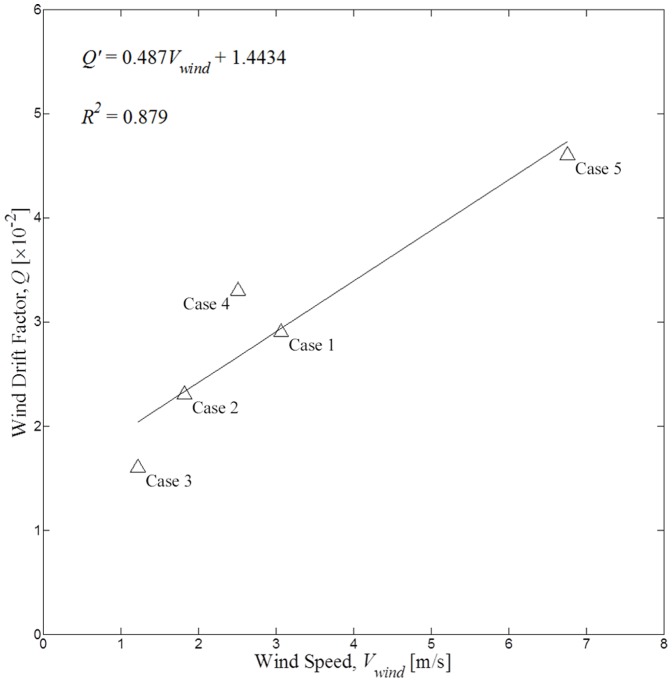
Scatter diagram of the wind drift factor and wind speed. Scatter diagram of the wind drift factor and wind speed for the cases 1–5 when the matching rates are optimum. The solid line is the regression line with the coefficient of determination *R^2^*.

Figure 9 shows the matching rate with the wind drift factor *Q*, and Table 3 shows the highest matching rates for all 5 cases. When the wind speed was relatively high (approximate mean speed: 6.76 m/s) for case 5, the matching rate as a function of *Q* showed large variation as can be seen in Figure 9. The highest matching rate was 76.3% with large standard deviation of 16.2%. The variation for case 1 is slightly larger than the rest, with the standard deviation of 7.5% and the highest matching rate of 94.6. The variations of matching rates for cases 2, 3, and 4 are similar with the standard deviations of 5.0, 5.8, and 6.8% and the highest matching rates of 71.3, 77.8, and 83.8% respectively. It can also be noticed from Table 3 that the wind drift factor increases with the average wind speed, and, as for the previous study based on satellite tracking buoy data [18], there appears almost a linear relation between them, as illustrated in Figure 10. It is then possible to take a further step to improve the simulation algorithm and increase the matching rate by using this linear regression equation for the wind-dependent drift factor. The newly modified empirical formula for the oil slick velocity is now defined as.
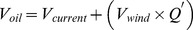
(3)where

**Table 3:** Mean wind speed, wind drift factor, and matching rate of simulation result compared with satellite images.

Simulation case	Mean wind speed [m/s]	Wind drift factor, *Q*	Highest matching rate
Case 1	3.07	0.029	94.64
Case 2	1.82	0.023	71.33
Case 3	1.22	0.016	77.81
Case 4	2.51	0.033	83.81
Case 5	6.76	0.046	76.28

Each mean wind speed was calculated from the data acquired at the AWS closest to the centroid of the slick area in each case.



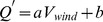
(4)The constants *a* and *b* are given by 0.4865×10^−2^ [s/m] and 1.4434×10^−2^ respectively, as shown in Figure 10. The result on the linear relation between the wind drift factor and wind speed is similar to that of [18] based on the satellite tracking buoy. However, the main differences are that, while the previous result was derived from the satellite tracking buoy data, the present result was obtained using multi-temporal two-dimensional satellite images, and that the oil slick movement was predicted using both the varying wind drift factor and the fixed drift factor of 0.03, and compared with satellite images. Care should be taken for these constants since they may well vary depending on locations and tidal conditions.

**Figure 11:**
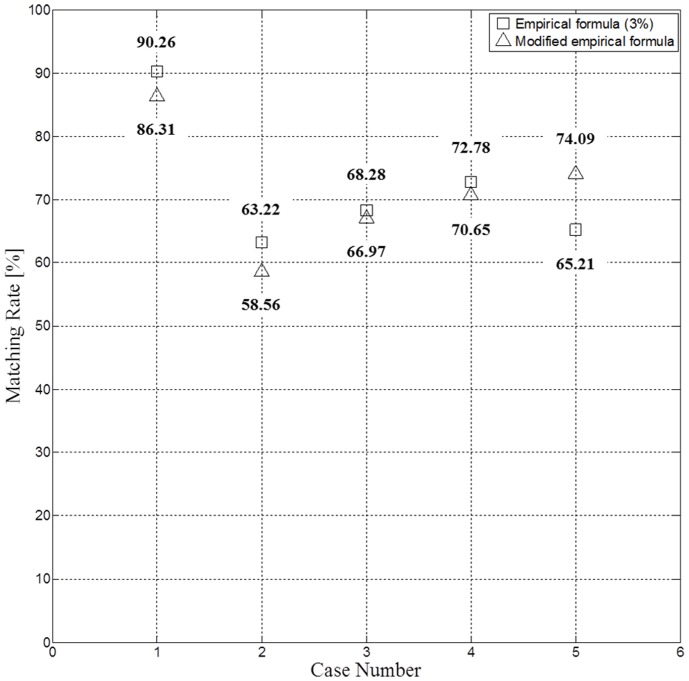
Comparison of matching rate. Comparison of matching rates (triangles) of the new model with those (squares) of the conventional model with a fixed wind drift factor of 0.03. The regression line for the new model was defined by equation (3) in the text.

It can be said that the new empirical formula is applicable for short-term (daily) prediction, because the wind drift factor in Table 3 was calculated as an average value for a predetermined period. Nevertheless, in order to test the validation of the new empirical formula, simulation was carried out using the model based on Equation (3), i.e., *V_oil_* changed according to *Q*’ as well as *V_current_* and *V_wind_* at every step of 30 minute intervals. The results were compared with those of Equation (1) with a fixed wind drift factor of 0.03 and the newly modified model with a varying wind drift factor as shown in Figure 11. The models with a fixed and linearly varying wind drift factors both showed similar matching rates for cases 3 and 4, while the matching rate of the new model decreased by 4–5% for cases 1 and 2 in comparison with those of the conventional model (they may be considered as being within statistical errors). However, an improvement of matching rate by 9% is obtained for case 5. The main reason causing this improvement may well be associated with the high wind speed (6.8 m/s) for case 5 in comparison with those (1.2–3.1 m/s) of other cases as can be seen in Figure 10. The validity of accuracy improvement based on a single data point may be questioned. However, the present study examined only 5 available cases, and only a single case had high wind speed to assess the validity. Considering the general trend of the previous results [18], [28] together with our result, it appears certain that there is an increasing trend of wind drift factor with increasing wind speed (although their results [18], [28] were based on the measurements by buoys), and Figure 10 and improved accuracy can be justified as a preliminary result. These results suggest that matching rates are influenced, to some extent, by wind drift factor in the study area characterized with strong tidal currents. Indeed, for the case of the 2011 *Deepwater Horizon* accident where the tidal variation is very small, Dietrich et al. [28] showed, using a similar transport model, that the best agreement with satellite data was obtained by ignoring the wind drift factor, i.e., *Q* = 0.0, where the tidal variation is very small.

It should be emphasized that the present model with new formulation does not take into account the fate effects such as evaporation, emulsification, dissolution and biodegradation, and therefore, it may not be suitable for long-term forecast of oil spill dispersion and movement. For example, the time difference for case 2 was 71 hours, longer than other cases (see Table 2), and the matching rate was lowest (see Table 3). At an early stage of oil discharge, oil thickness and spread are dominant factors, and evaporation and weathering become important at later stages [4], [5]. We focused our simulation of oil movement within 7 days of the accident, and the information on the input oil particles is updated by successive satellite data. These factors, therefore, might have minimized the errors associated with initial and latter stages.

Finally, mention should be made on the studies of Abascal et al. [18] and Dietrich et al. [28] who used similar Lagrangian algorithms composed of the advection and horizontal turbulent diffusion. The difference optimal wind drag coefficient, i.e., wind drift factors were estimated according to characteristics such as tidal currents and bottom topography at each position. It may be interesting to take into account the tidal conditions and geological characteristics at each location for the models in the future study.

## Conclusions

The fixed wind drift factor may cause uncertainty for prediction results of oil slick movement using the empirical formula. In this study, the effect of the wind drift factor was investigated for the case of the 2007 *Hebei Spirit* accident under strong tidal condition by comparing the results of numerical simulation with 5 sets of satellite image data. It was found that the best matching rates were obtained with different wind drift factors, and that the optimum drift factor is, to first order, linearly proportional to the wind speed. Based on the regression relation, a modified empirical equation was proposed, and using this equation the movement of oil slicks was re-calculated by numerical simulation. Comparison of the results with the satellite data showed high matching rate of over 60%, in particular, the new model improved the matching accuracy by 9% under strong wind speed and high tidal condition in comparison with the conventional model. For more stringent validation, further study is required with more data and other location which has difference conditions, such as bottom topography, dominant surface current, etc., since the present model was derived from the limited data as a preliminary result.
